# Wood ants learn the magnetic direction of a route but express uncertainty because of competing directional cues

**DOI:** 10.1242/jeb.244416

**Published:** 2022-08-26

**Authors:** Thomas S. Collett, Andrew O. Philippides

**Affiliations:** 1 School of Life Sciences, University of Sussex, Brighton BN1 9QG, UK; 2 School of Engineering and Informatics, University of Sussex, Brighton BN1 9QJ, UK

**Keywords:** Ant navigation, Magnetic compass cues, Path integration, Route learning

## Abstract

Wood ants were trained indoors to follow a magnetically specified route that went from the centre of an arena to a drop of sucrose at the edge. The arena, placed in a white cylinder, was in the centre of a 3D coil system generating an inclined Earth-strength magnetic field in any horizontal direction. The specified direction was rotated between each trial. The ants’ knowledge of the route was tested in trials without food. Tests given early in the day, before any training, show that ants remember the magnetic route direction overnight. During the first 2 s of a test, ants mostly faced in the specified direction, but thereafter were often misdirected, with a tendency to face briefly in the opposite direction. Uncertainty about the correct path to take may stem in part from competing directional cues linked to the room. In addition to facing along the route, there is evidence that ants develop magnetically directed home and food vectors dependent upon path integration. A second experiment asked whether ants can use magnetic information contextually. In contrast to honeybees given a similar task, ants failed this test. Overall, we conclude that magnetic directional cues can be sufficient for route learning.

## Abstract (precis)

**Summary:** Wood ants can learn and remember overnight the direction of a short foraging route that is specified magnetically.

## INTRODUCTION

Ants and bees can guide their paths during navigation by magnetic cues (for reviews, see Wajnberg et al., 2010; Wiltschko, 2012; Fleischmann et al., 2020). An excellent example comes from the desert ant *Cataglyphis noda* (Fleischmann et al., 2018): young *C. noda*, before their sun compass is calibrated, rely on magnetic cues to provide compass information during their learning walks when they first leave their nest and are naive to the world outside it (for review, see Zeil and Fleischmann, 2019). During the walks, ants loop around the nest, periodically turning toward it, giving themselves the opportunity to memorise views that can guide their later returns to the nest. Manipulation of the direction of the magnetic field during normal outdoor learning walks reveals that turns to face the nest are directed by path integration, with the Earth's magnetic field providing compass information. Desert ants do not seem to rely on magnetic information in later life and switch to a time-compensated sun compass that may give more precise directional information (e.g. Wehner and Müller, 2006).

The wood ant *Formica rufa* is known to be sensitive to the Earth's magnetic field (Çamlitepe and Stradling, 1995). We examined whether these ants can remember the magnetic direction of a route to a food site. The question whether route learning can be accomplished when ants must rely upon magnetic cues has to our knowledge not been tackled previously. It is valuable to know the extent to which magnetic cues are, in this respect, on a par with celestial cues to direction.

To assess the ants’ ability to learn and recall a magnetically specified direction, they were trained to reach food at a point on the circumference of a small arena in a 3D 1 m^3^ magnetic coil system. To prevent other potential directional cues from being persistently reinforced, the chosen magnetic direction was rotated after each trial.

One problem in interpreting the ants’ behaviour is that they rarely sustain a straight path to the food. This difficulty is perhaps not surprising. A low signal-to-noise ratio of magnetic information seems to be a general feature among animals (Johnsen et al., 2020). Consequently, magnetic information may be rendered uncertain by competing cues to direction (Dreyer et al., 2018; Johnsen et al., 2020), as also happens in our ants. The most useful measure of the ants’ performance that we found is their facing direction relative to the food at the start of their trajectory. This information can tell us whether ants learn the magnetically specified direction from the start to the food. But it does not reveal whether ants can regain their route if they wander off it, i.e. whether the ants have ‘food vectors’ and also home vectors that rely upon path integration operating through magnetic cues. We approached this question with a different analysis, which is detailed in the Results.

The ants’ apparent difficulty in following the magnetically signalled direction during tests also occurs during training. In some training trials, especially at the start of training, the food site was often indicated by a vertical, black bar placed on the arena wall just above the food. Not infrequently, ants headed in the opposite direction from the bar. In tests too, ants can express their uncertainty by moving briefly or for longer periods in the opposite direction from the food toward the centre of the arena or toward a fictive goal at the diametrically opposite point at the edge of the arena.

A second series of experiments explored whether magnetic cues to direction can provide a contextual cue that enables ants to select between two routes. Ants were released at the centre of the same arena and were confronted with an upright and an inverted triangle in fixed positions on the arena wall. Sucrose was placed at the bottom of one of the triangles depending on the orientation of the magnetic field in the arena. Previous work on ants and honeybees suggests that ants might be able to solve this problem. They distinguish between these shapes and can be trained to approach either of them (Judd and Collett, 1998). Moreover, honeybees can be trained to use magnetic direction to decide which of two patterns they should choose (Frier et al., 1996).

Finally, to anticipate our results, we find that wood ants are able to learn and recall routes when they must rely on magnetic cues for their direction, and we provide suggestive evidence that path integration can also operate with magnetic cues.

## MATERIALS AND METHODS

### Ants

Experiments were performed on three colonies of laboratory-maintained wood ants, *Formica rufa* Linnaeus 1761, two in 2018 and one in 2019. All the colonies were collected from Broadstone Warren, East Sussex, UK. Initial experiments, from January to March 2019, were on 2018 colonies that had been in captivity for at least 6 months. In the event, these initial experiments turned out to be a training experience for the experimenter, rather than for the ants. Experiments on the 2019 colony took place between June and August, a few weeks after the colony was taken to the laboratory. All the presented data come from this colony. The colonies were kept under a 12 h:12 h light:dark cycle and were sprayed with water daily. Water and sucrose dispensers were always available, except during experiments, when the colony was given limited access to sucrose to encourage enthusiastic foraging. Crickets were supplied several times a week. Before training, about 30 ants were marked individually with coloured enamel paint (Testor) and about a third of this group completed training.

Experiments were performed in an artificially lit laboratory with some natural light from a window distant from the arena. The arena was lit directly by a ring of white LEDs fixed above a 25 cm diffuser (Arlec electrical ceiling light) and placed centrally above the magnetic coils.

### Controlled magnetic field in the experimental arena

**Fig. 1.**
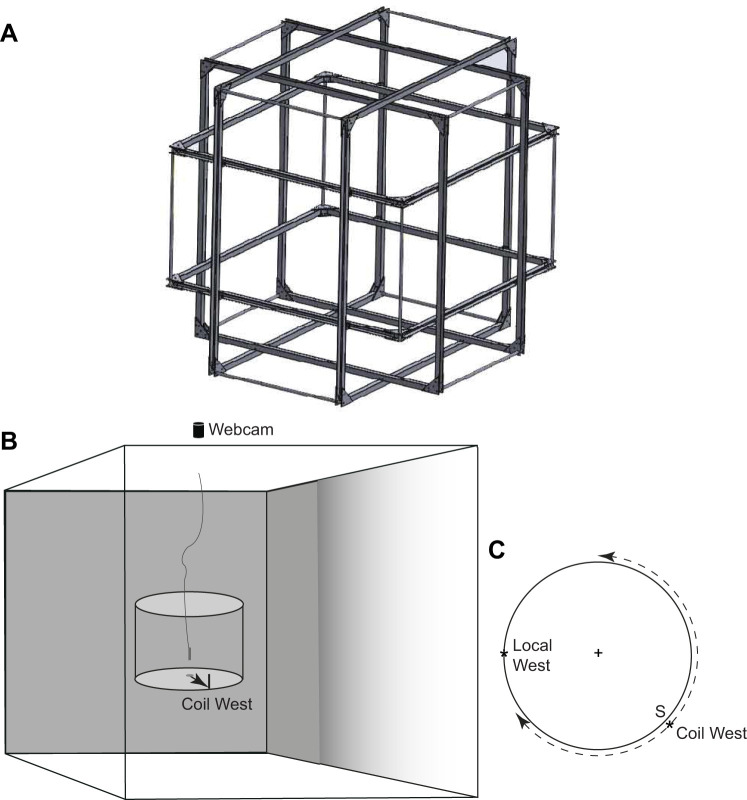
**Magnetic coils and experimental set up.** (A) Diagram of the 1 m, 3D coil system (provided by Stephan Eder, claricent). Paired coils are in a Helmholtz arrangement. (B) Illustration of the arrangement of the cylinder and arena floor in the centre of the coil system. The direction of the food on the arena floor is shown by an arrow pointing at coil West. The black bar and sucrose during training were placed at the edge of the arena at the point of the arrow. The ant was released at the centre of the arena by raising the cylinder (shown a little above the arena) from the grooved base plate by means of a nylon thread. The webcam was positioned above the frame and the LED lights (not shown) were above the webcam. (C) Top view of the cylinder showing coil West and local West. The dashed arrows illustrate the two positions to which coil West could be rotated on the next trial.

Three pairs of single-wrapped 1 m-diameter coils arranged in a cube (claricent: info@claricent.de), generated a uniform Earth-strength inclined magnetic field (calibrated for London) within a volume of about 50 cm^3^ (Fig. 1A). The coils give a uniform magnetic direction within 5% for 45–50 cm in each direction and within 1% for a 25–30 cm cube. Three computer-controlled power supplies (Tenma Model 72-2685 Digital Controlled DC Power Supply) and power amplifiers (constructed in house) determined the magnetic direction within the central cube in 5 deg steps. On each trial, the set directions were confirmed with a magnetic compass. No change between the setting command and the consequent magnetic reading was detected over the course of the experiments.

### Route training

A white painted cylinder (38 cm diameter, 32 cm height) with its bottom removed was placed in the centre of the platform in the centre of the coil system (Fig. 1B). Ants taken from their nest were released in the centre of the circular arena at the bottom of the cylinder and could find a drop of sucrose at the edge of the arena by travelling west from the arena centre (Fig. 1B,C). After every trial, the magnetic direction (coil West, Fig. 1C) was rotated by about 100 deg relative to the laboratory. The direction of rotation switched between clockwise and anticlockwise every day. On some trials during training and tests, the coils were turned off and the ants navigated toward ‘local West’, i.e. the reading of the compass when the coils were inactive (Fig. 1C). To minimise the use of trail cues, the paper on which the ants walked was shifted relative to the bucket between trials, or reversed, or replaced.

Training was in two stages. In the first stage, individually marked ants taken from the nest were trained in groups of 5–6 individuals. A group of ants was put in a small release cylinder in the centre of the arena. The cylinder was 7 cm wide and 5 cm high with a 30 deg exit in the wall that pointed in the direction of the sucrose. At the start of training and periodically through training, but not in tests, the magnetic direction to the sucrose was reinforced by a black vertical bar (6 cm high by 0.5 cm wide) held by Blu Tack to the inner wall of the cylinder just above the sucrose (Fig. 1B).

In the second stage of training, marked ants were released individually in the centre of the arena from a portable release compartment (Fig. 1B). The device consisted of two parts: (i) a Perspex cylinder (2.7 cm high, 2 cm wide) with an open bottom and closed top; (ii) a Perspex circular base plate (5 cm wide) with a circular groove in its centre that was cut to accept the open end of the cylinder. A length of nylon fishing line was attached to the top of the cylinder. An ant was placed inside the cylinder with the open end facing upwards. The base plate was then put on top with the cylinder slotted into the groove, after which the release compartment was placed right-side up in the centre of the arena in no particular orientation. The fishing line was looped over a support 58 cm above the arena so that the cylinder could be pulled up vertically to a level just above the recording video camera and then secured, thereby releasing the ant. This method frees the ant to move in any horizontal direction. On a few occasions, a trial was aborted because the ant clung to the inside of the cylinder and was raised with it.

As training progressed, the bar was removed on many of the trials. If an ant failed to reach the sucrose when there was no bar, the bar was replaced to ensure that the ant was rewarded. Because the arena is so small, it is difficult to exclude the possibility that, during training, ants are attracted directly to the drop of sucrose by cues emanating from it. Consequently, training trials were only analysed to examine paths in which the ants travelled directly away from the goal.

### Video recording

An HD webcam (Microsoft 6CH-00002, 1200×1080 pixels), which surveyed the whole arena from a position just above the coils, recorded the ant's path at 30 frames s^−1^ from the moment the cylinder was raised above the base plate. Paths were stored on computer as MP4 files that were later re-coded as AVIs and then processed with custom-written code in MATLAB to extract both the position and facing direction of the ant. These data were checked and corrected by hand using methods described in de Ibarra et al. (2009).

### Tests

Once ants were trained, nine test trials were given with no bar and no sucrose. To see whether ants have a ‘longer’ term memory of the magnetic direction, four of these tests were given at the start of the experimental day, before there had been any training (referred to as ‘early’ tests). Tests were separated by a varying number of training trials (see Table 1), with no intervening training trials when tests were given at the end of one experimental day and the start of the next day. Before each test, the cylinder and magnetic field were rotated in opposite directions and the paper surface under the cylinder was changed or turned over to make sure that there were no traces of odour or sucrose. During training and test trials, not all ants in the cohort could be found on the surface of the nest. We therefore limited our analysis of the data during tests and training to the trajectories of 9 ants that appeared for at least six of the nine tests that were given.

**Table 1.**
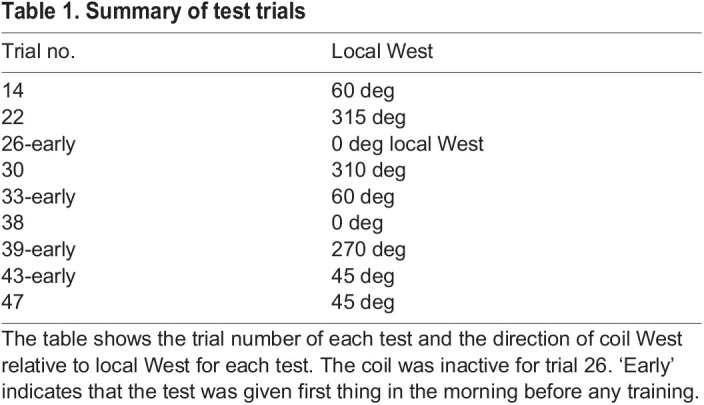
Summary of test trials

### Analysis of tests

Because it became clear that ants do not sustain their paths, we analysed the facing direction of the ant's body relative to the line from the centre of the arena to the goal (body angle relative to 0 deg). We examined body angle over time in successive intervals of 2 s from the start of a test. Data extraction and selection used MATLAB scripts developed by A.O.P.

The figures show the data accumulated in various ways in 10 deg bins. Statistics were performed on the values of the modal bins of single test trials. Circular statistics are calculated in MATLAB (Berens, 2009). We used the *V*-test with a predicted value of 0 deg body angle to see whether distributions have a mean direction. We also obtained the circular mean, *R*, the resultant vector length, and the circular standard deviation.

In order to determine whether ants have food or home vectors, we extracted episodes in which ants fixated the food or the start point in the centre of the arena for at least 7 frames within ±10 deg. Fixation is defined as the facing direction being within ±10 deg of the goal position (food or start). As the heading direction is a bit variable, we allowed a single frame within the 7 frame episode to be outside the ±10 deg limit. We then examined the body angles of these fixations relative to 0 deg.

### Magnetic direction as a contextual cue

A second experiment was conducted with newly trained groups of ants. Ants were released in the centre of the arena as already described. They chose between approaching an upright or an inverted triangle on the cylindrical arena wall to find sucrose at the bottom of one, depending on the orientation of the magnetic field. The sucrose was beneath the upright triangle, when the direction from the central start point to the upright triangle was West. The inverted triangle was rewarding when the magnetic direction to that triangle was North. The triangles (7.8 cm high, 6.3 cm base) were cut from black card. They were fixed in position, 90 deg apart with their tip or base on the arena floor. The ants’ paths were recorded during training and test trials following the same procedure as in the route-learning experiment.

## RESULTS

We show below that ants learn the magnetic direction of a route from a start point to food and that they are also likely to compute food and home vectors. Their behaviour is somewhat complicated. The likely reason is that other directional cues interfere with the expression of the ants’ magnetic knowledge. We start by investigating the ants’ uncertainty about using magnetic information during training trials with food. We then analyse test trials in which food is missing.

### Route learning

#### Training trials with visual cues

**Fig. 2.**
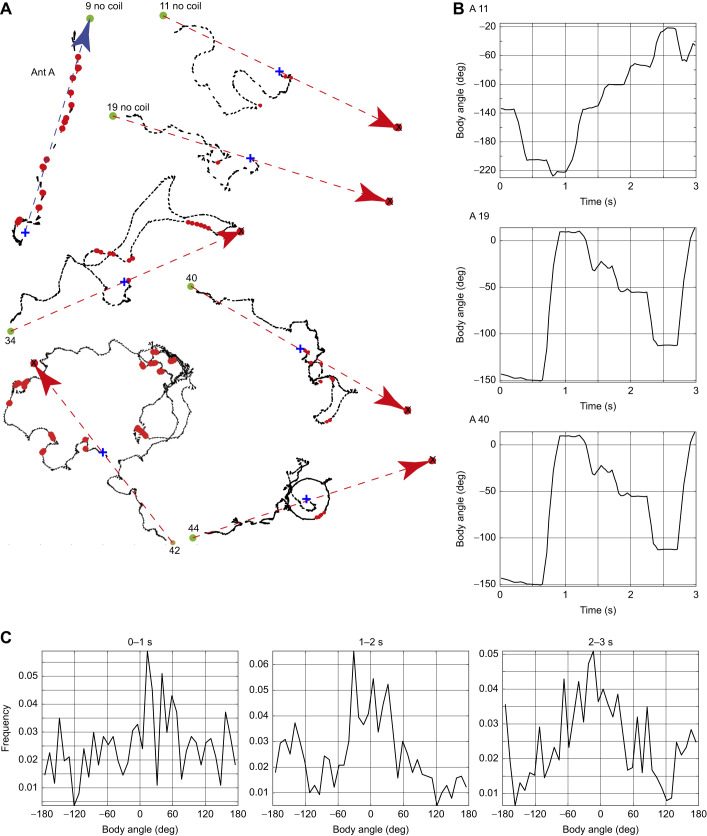
**Training trials with magnetic direction supported by a black bar.** (A) Each panel shows the path of ant A on a training trial. Blue crosses mark the starting point. Red circles in the trace of the ant's path indicate frames in which the ant faced (within ±10 deg) either toward the food (marked with a blue arrow pointing at a green circle, trial 9) or toward a fictive goal in the opposite direction (red arrow pointing at a red circle with a cross, all other trials). The real and fictive goals were both 18 cm from the central starting point. Numbers by the food position indicate the training trial. In trials marked ‘early’, i.e. early in the day, the coil system was off and the ant was guided by the natural magnetic field. The circle surrounding the start in trial 44 is the ant circling around the base of the release chamber. (B) Three individual plots of body angle during the first 3 s of a trial. (C) Frequency plot of body angle over the interval 0–3 s. Data come from 49 training trajectories with the black bar in place.

During training trials in which the ant can be guided to the sucrose by the black bar and by magnetic cues, the ant usually reached the food (e.g. Fig. 2A). We found, unexpectedly, that, at the start of their approach to the sucrose, ants often faced and moved in the opposite direction. This effect is illustrated by the seven training trials from one ant in which the bar was present. Trial 9 was the only one in which the ant travelled directly toward the bar and the food. In trial 11, the ant left the start and moved for a short stretch in the opposite direction. In trials 34, 40 and 42, the ant went all or most of the way to a fictive goal in the diametrically opposite position to the real goal. In trials 19 and 44, the ant's intentions in the first phase were not clear. Similar examples of travel in the opposite direction occurred in other ants (Fig. S1). This behaviour cannot be attributed to some quirk in the applied magnetic field (coil West) as it also occurred in three trials at the start of the day (trial 11 in Fig. 1; 2 further cases in Fig. S1) when the coils were inactive, and the ants were guided by the Earth's natural magnetic field (local West).

To assess when during the start of the trajectory ants most frequently switched to facing toward a goal in the opposite direction, we analysed the 49 training trials with a bar that were performed by the 9 focal ants. The orientation of the ants’ body was accumulated over 1 s intervals for the first 4 s of their paths. In all plots (Fig. 2C), the major peak was along the line from the start to the goal. During the first 2 s there were also subsidiary peaks at around ±150 deg. In the third second (2–3 s), the largest subsidiary peak was at 180 deg. This subsidiary peak had disappeared by the fourth second (not shown). The ants’ peak distance from the start during the third second was close to 3 cm.

#### Tests

##### Facing angles

Eight of the tests were given with coil West pointing in a variety of directions relative to local West (Table 1).

Test 26 differed in that it was guided by local West. During these tests, ants also faced toward the real and fictive goals.

**Fig. 3.**
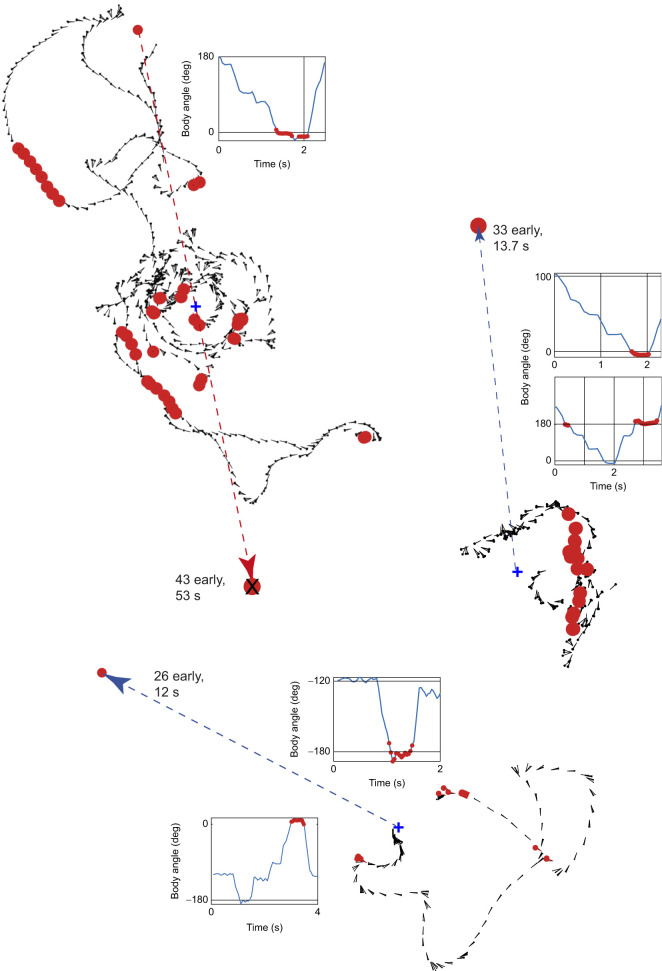
**Trajectories of three tests of ant E.** Each panel shows all or part of a path recorded during one test, with the duration of the excerpt given below the test number. The ant's path during the trajectory is shown every 33 ms by a black line indicating the ant's body angle, with its head represented by a dot. Plots of body angle over time give the ant's facing direction (body orientation) relative to the magnetically defined goal, with 0 deg or 180 deg indicating the direction of the real (0 deg) or fictive (180 deg) goal. Red circles indicate when the facing direction was within ±10 deg of the real or fictive goal. The start of the path was at 0 s. ‘Early’ indicates that the test was given before any training on that day.

We first illustrate this behaviour through plots of body angle during the initial stretch of two of the nine tests taken by ant E (Fig. 3). Goal facing often occurred within the first few seconds of a test. Moments in which the ant's body was oriented toward the real or the diametrically opposite fictive goal (±10 deg) are shown in red in Fig. 3. These points can occur at a trough or peak of the plot of body orientation. Ants at troughs or peaks may have reached a point of decision about where to face next and so switched between the two goals. The details of these two facing periods are clarified in two plots shown at different time scales in Fig. 3: the ant faced both goals in tests 26 and 33. The whole of test 43 is also shown (Fig. 3), as a rare example of the ant reaching the goal after a tortuous approach.

**Fig. 4.**
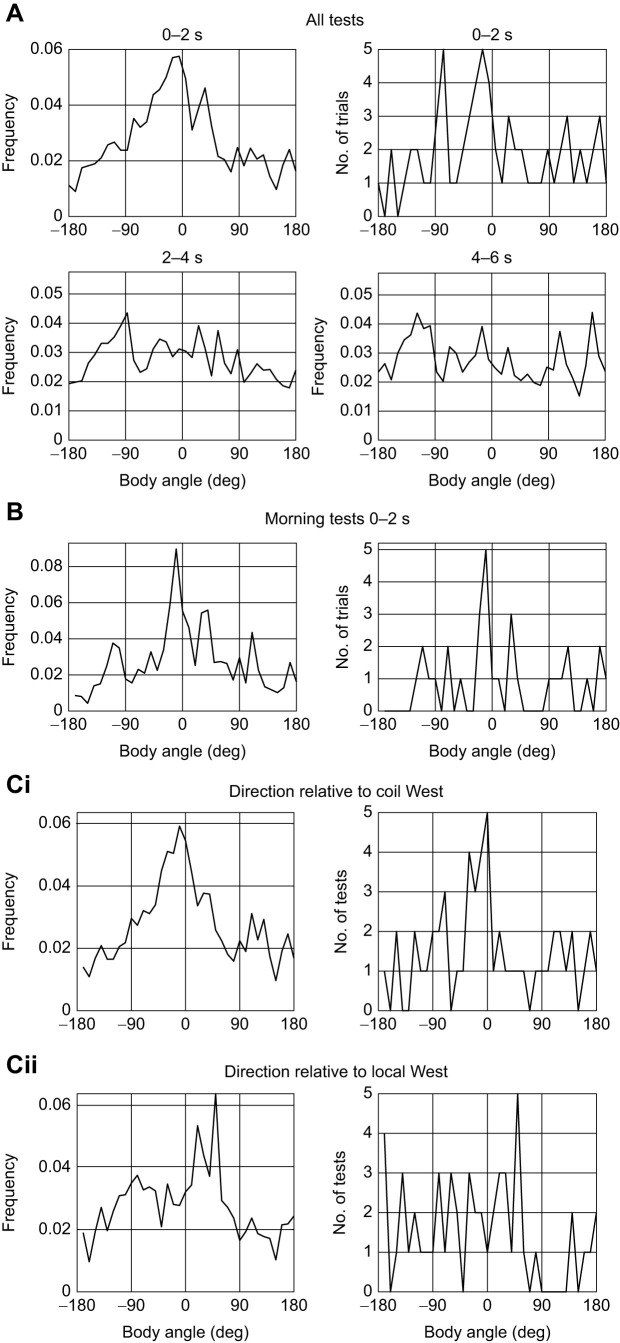
**Body orientation in tests relative to coil West or local West.** (A) Top: frequency of body angles relative to coil West of all 69 tests of 9 ants during the first 2 s of each test (left) and the same data giving the peak modal value for each of the 69 tests (right). Bottom: frequency of body angles for later time intervals (2–4 s and 4–6 s). (B) Body angles during early morning tests over the 0–2 s interval. Data plotted as in A (top). (C) Body angles plotted (i) relative to coil West and (ii) relative to local West or to room cues. Plots exclude 17 of the 69 tests in which coil West coincided with local West. Data are plotted as in A (top).

All the tests of all 9 ants gave a total of 69 test runs. To examine the group behaviour of the ants during these tests, we analysed body angles in blocks of 2 s starting at the beginning of each test. From 0 to 2 s, the ants tended to face toward coil West (*V*-test, *V*=13.08, *P*=0.013, *n*=69; Fig. 4A; circular mean and s.d. −12.2 and 72.7 deg and vector length *R*=0.19). For other 2 s intervals up to 8–10 s (e.g. Fig. 4B), the facing angles were widely spread with no interval in which the ant faced in a specific direction toward or away from coil West.

The subset of 32 tests given early before any training were initially similar to the full set. From 0 to 2 s, ants faced close to coil West (*V*-test, *V*=7.71, *P*=0.025, *n*=31; Fig. 4B; circular mean and s.d. 10.65 and 70 deg and vector length *R*=0.25), indicating that the ants’ memory of the magnetic direction from the start to the food endured overnight. But there was a difference in the ants’ behaviour over later 2 s intervals: the peaks first rotated anti-clockwise followed by intervals with three clear peaks. The distribution of all the peaks had three rough clusters: toward the food, in nearly the opposite direction and perpendicular to the food-nest direction (Fig. S2). To examine how performance varied between ants and across tests, the frequency of body angles during the 0–2 s interval is shown separately for each of the nine test trials and for each of the focal ants in Fig. S3. Tests appear to become better over the course of the experiment and some ants (e.g. E, L, T, U) performed more precisely than others.

Lastly, we asked whether room cues play a role over the 0–2 s interval. To do so, we excluded tests in which coil West was the same as local West. Body angles of this depleted sample are plotted relative to coil West and local West in Fig. 4C, showing a peak at zero for the coil West plot (Fig. 4Ci) and a displaced peak for local West (Fig. 4Cii). Despite the peak in the accumulated data (Fig. 4Ci, left) appearing clearer than the peak for local West (Fig. 4Cii, left), the reduced data were not significantly directed toward the specified magnetic direction when taking each path independently (*V*-test, *V*=7.93, *P*=0.062, *n*=53). But, at a low level of significance, they were directed toward a room cue (*V*-test, *V*=9.02, *P*=0.040, *n*=53). The vector lengths were *R*=0.16 for magnetic cues and *R*=0.19 for room cues. Circular mean and s.d. were −17.1 and 74.4 deg (*n*=53) for magnetic cues, and −28.0 and 72.8 deg (*n*=53) for room cues.

##### Food and home vectors?

Although initially ants tended to face along the magnetically specified direction, thereafter their direction often wandered, with occasional episodes in which they faced in the direction of the food. Do these episodes indicate that the ants are guided by a food vector through path integration (Dyer et al., 2002)? A food vector is equivalent to a home vector but instead of ants monitoring their distance and direction from home, they monitor the distance and direction of a food source from home and move to reduce the magnitude of the vector. They can do that from any point along a more circuitous path, provided that path integration is operating from the start. Here, we asked whether ants face the food and the start point in a manner suggesting that they have food and home vectors.

Two criteria must be met to infer that facing the food when distant from the line between the start and the goal is a reliable sign of the use of a food vector or that facing the nest indicates the use of a home vector. The first is that facing the food or nest occurs over a considerable period. We analysed instances of food and nest facing (±10 deg) that persisted for at least 7 consecutive frames (230 ms). The second is that the body angle when facing these points differs substantially from 0 deg, indicating that the ant is relatively distant from the line connecting the start to the food and so is not merely facing in a remembered direction.

**Fig. 5.**
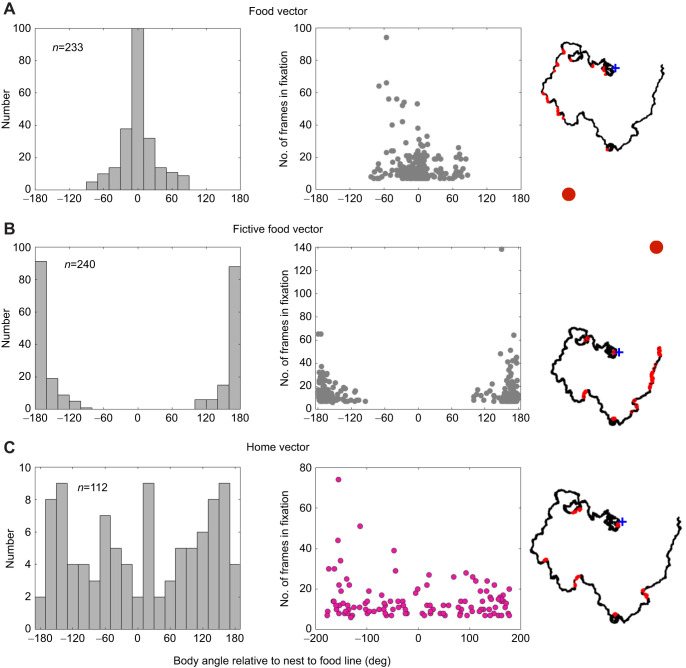
**Evidence suggesting that ants compute food and home vectors.** (A) Fixations of food point. (B) Fixations of fictive food. (C) Fixations of start point. Left: histograms of body angle when ants face positions of food, fictive food or nest in tests with food absent (20 deg bin width). Middle: number of frames in each fixation of the left column plotted against body angle of that fixation. Right: trajectory of ant Q in test 39. Its fixations when facing food, fictive food or nest are shown in red and are distributed throughout the trajectory. Blue crosses indicate the start point. Red circles show the position of the food or fictive food.

To see whether these criteria were fulfilled, we extracted all instances of food, fictive food and nest fixations (Test 47 was excluded because of the atypical training trials before that test). These fixations can occur throughout the trajectory as might be expected for an expression of path integration and were often much longer than 7 frames (Fig. 5). The ant's body angle in the mid-point of each fixation was measured relative to the line between the start point and the food (Fig. 5). The resulting distributions of body angles during fixations of the food and fictive food had peaks at 0 deg and 180 deg. The spread away from the peak was larger when facing the food than when facing the fictive food. Moreover, fixations away from the peak tended to be longer when facing the food than when facing the fictive food. This difference suggests that there may well be a food vector, but that ants are less likely to compute a vector to the fictive food. The distribution of body angles in the ants’ fixations of the start point was broad, strongly suggesting the presence of a home vector, as occurs in *Cataglyphis* (Fleischmann et al., 2018).

#### Room cues to direction

**Fig. 6.**
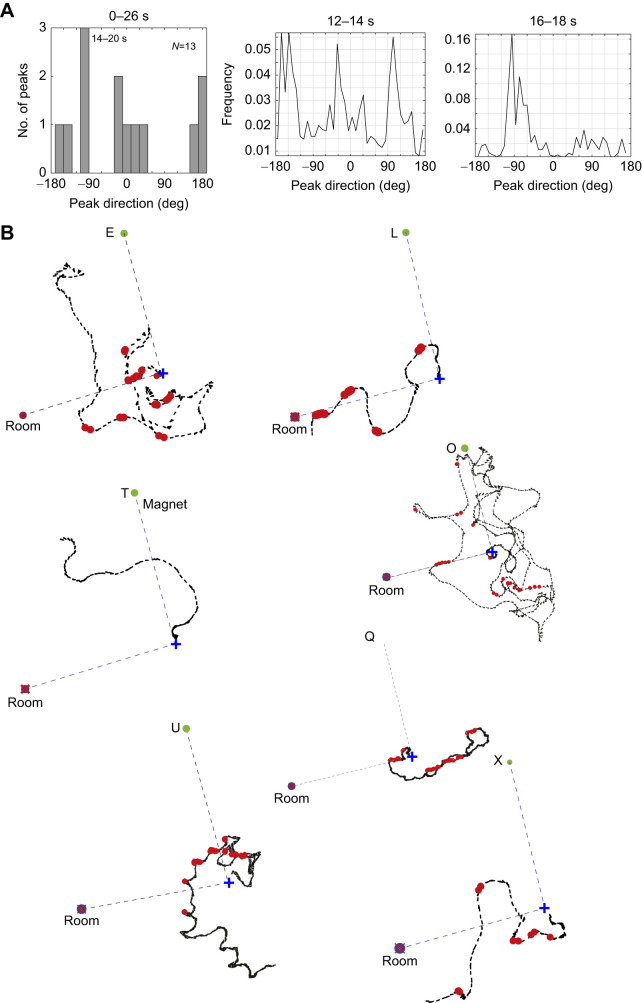
**Test after three consecutive training trials with food in the same direction relative to the room.** (A) Left: histogram of peak body angle sampled every 2 s during the first 26 s of the test. For the three intervals covering 14–20 s, the ants’ peak direction was perpendicular (−90 deg) to the centre to nest direction. Right: frequency of body angle in the intervals just before (14–16 s) and during which (16–18 s) ants faced at −90 deg to the test direction. (B) Individual trajectories of all tested ants (E, L, O, Q, T, U and X). Previous training direction is indicated by a marker at the goal distance labelled ‘room’. Red circles show when the ant faced the marker within ±10 deg. Other details as in Fig. 2.

The results so far emphasise that, in addition to facing in the goal direction, ants can face and move in the opposite direction during training and test trials. One of several possible reasons for this behaviour is the presence of competing directional cues that are fixed to the room, rather than to the rotating magnetic direction. It is, for instance, unlikely that the illumination of the arena was uniform. To examine the possibility that there are competing directional cues, we ran a test at the end of the experiment [data from this test (test 47) are also included in Fig. 4 and Fig. S3]. Before the test, ants had three consecutive training trials in which the magnetic field was in the same direction relative to the room and to any cues associated with it. In the test that followed, the magnetic direction was rotated 90 deg clockwise from the training direction. At the start of the test (0–2 s), ants as a group faced toward coil West (Fig. S3). Later on (14–20 s), their body angle had rotated by 90 deg in the direction of the three prior training trials (Fig. 6A). Interestingly, just before they faced in the previous training direction (14–16 s), the ants had a prominent directional peak at 180 deg, perhaps indicating some uncertainty of the correct direction to follow.

The group's behaviour was reflected in the ants’ idiosyncratic paths (Fig. 6B). Points in which an ant faced in the room-specified direction (±10 deg) are marked by red circles in Fig. 6B. Ants E and Q moved in this direction at or near the start of their paths. After the initial stretch, ant E's path became more erratic, until at the end, the ant moved in the magnetically specified direction. Ant Q's subsequent path was first directly away from the room-specified goal and then directly toward it. Ant L began with a stretch in the magnetically defined direction, after which it nearly reached the room-specified goal position. The bulk of ant O's path was along the magnetically specified axis, with some stretches in which it faced in the room-specified direction. Ant X began by moving away from the magnetically specified goal. It then moved toward the room-specified goal before wandering off. Ant T began with a short stretch toward coil West, and ignored the room-specified goal. Ant U's intentions were unclear; it took a semi-circular path around the nest, during which it briefly changed direction and faced toward the room goal. Thus, 4 (E, L Q, X) out of the 7 tested ants had clear path segments towards the room-specified goal. Though insufficient as proof, these results suggest that the ants may be unwilling to rely entirely on magnetic cues and are eager to find other more reliable directional cues (Johnsen et al., 2020).

### Do ants use magnetic direction as a contextual cue?

**Fig. 7.**
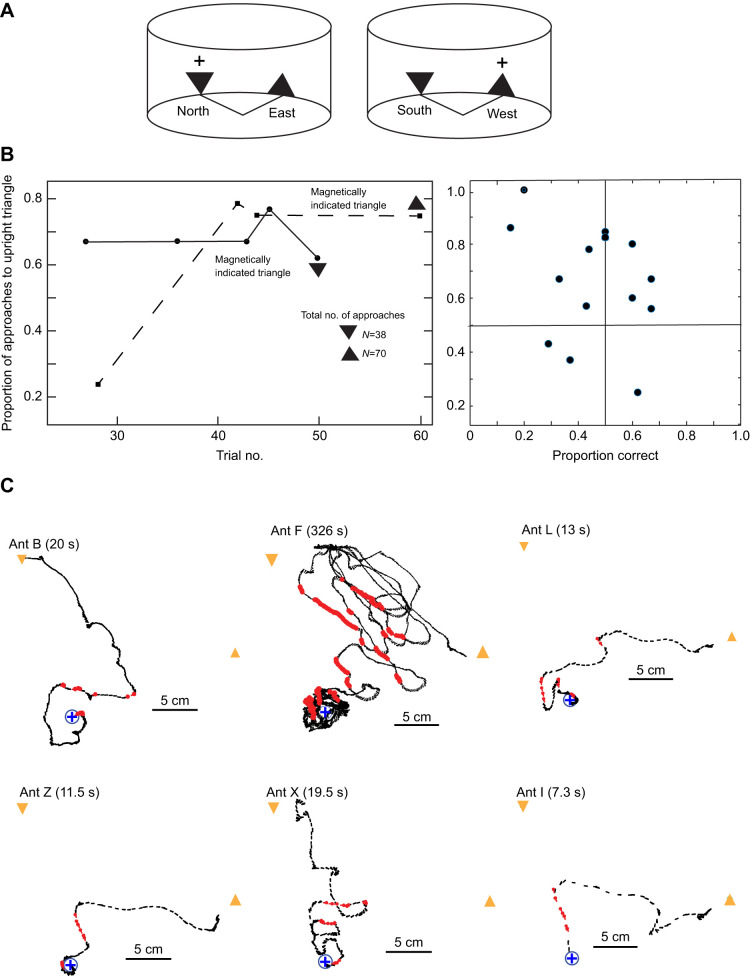
**Training to two triangles.** (A) Schematic diagram of the training arrangement showing magnetic directions from the centre of the arena to the base or apex of the triangles that were fixed in one place to the inner face of the cylinder wall. The cross shows which triangle was rewarded for each arrangement. (B) Left: proportion of trials (training and tests) in which the upright triangle was reached first. The dashed line indicates trials where the upright triangle was rewarded; the solid line indicates trials where the inverted triangle was rewarded. Right: each of the 14 dots represents the performance of an individual ant. The position of the dot gives the proportion of trials in which the upright triangle was reached versus the proportion of correct trials. (C) The trajectories of 6 out of 13 ants that appeared for trial 60 – a training trial. The position of the triangles is shown in yellow. The trajectory is coloured red when ants faced one or other triangle within ±10 deg.

In this experiment, different groups of ants were again released at the centre of the arena. They were confronted with an upright and an inverted triangle in fixed positions, 90 deg apart, on the cylindrical arena wall with the base or apex close to the floor (Fig. 7A). When the magnetic direction from the central start point to the upright triangle was North, sucrose was placed below the upright triangle.

Sucrose was below the inverted triangle when the magnetic direction to that triangle was West (Fig. 7A). Thus, ants can only learn the task if they can appreciate that there is a linkage between the vertical orientation of a triangle and the direction of the magnetic field from the centre of the arena to that triangle.

Despite many training trials, ants did not learn to approach the correct triangle. During the course of training and testing, individual ants were recorded in 10 trials between trials 27 and 60. For each recorded trial, we noted which triangle the ants reached first, ignoring the two times in which an ant failed to reach either triangle. We then plotted the proportion of ants approaching the upright triangle (Fig. 7B, left). Irrespective of which triangle was signalled, ants tended to go to the upright triangle, with no improvement during training.

The performance of individual ants was assessed using 14 individuals that were recorded on five or more of the 10 trials. For each ant, we plotted the proportion of trials in which the ant first reached the upright triangle against the proportion of trials in which the ant first reached the correct triangle: 9 out of 14 ants chose incorrectly on half or more of their recorded trials; 11 out of 14 ants preferred to approach the upright triangle.

The ants’ behaviour is illustrated through individual paths taken from the last training trial (60) in which the upright triangle was rewarded (Fig. 7C). Whichever triangle the ants reached first, they tended to approach the non-chosen triangle as well. Ants sometimes just switched their path from one triangle to the other (ants B, Z, I). At the extreme, they oscillated between the two triangles over several cycles (ant F). The ants in both their first and last recorded trials always switched over ca. 2 s from facing one triangle to facing another. They did not turn to face directly away from a triangle (Fig. 7C). Despite this indecision within a trial, the ants were nonetheless strongly biased in favour of reaching the upright triangle first. This bias could be because the upright triangle is more ecologically plausible, and the visual system is better tuned to it.

In another experiment of the same kind with a different set of ants, the triangles were placed 180 deg apart. In this case there was a weaker but just significant preference for the upright triangle (inverted triangle 17/59 correct choices, upright triangle: 22/41 correct choices, two-tail Fisher exact probability test: *P*=0.014). Again, there was no sign that ants learnt to choose the correct triangle.

## DISCUSSION

This study provides evidence that ants can learn and remember overnight the magnetic direction of a route between a starting point and a feeding place. Behavioural signs of this memory were clearest at the very start of the route. In addition, there is suggestive evidence that the ants compute food and home vectors, indicating that path integration can function with direction indicated by magnetic cues (Fleischmann et al., 2018).

At least two factors could contribute to the lack of a direct path to the food. One is the small area over which magnetic direction could be reliably manipulated. At the start of normal routes in larger spaces, the direct route vector and desired compass direction coincide for a while. In a small arena, slight deviations from the route will cause the direction of the route vector to diverge from the specified magnetic direction, perhaps confusing the ant over the correct path to follow.

A second possible reason for leaving the magnetically defined route is the existence of competing cues from other directional signals (Fig. 6). The ants may be unsure whether they should move in the specified magnetic direction or follow another cue. The ants’ uncertain path to food combined with a likely ability to integrate their path and generate food vectors suggests some independence between path integration and the factors contributing to the ants’ uncertainty.

A comparison between the ants’ behaviour during early morning tests before training and that in later tests supports the suggestion that following magnetic cues can be disturbed by competing cues (Dreyer et al., 2018; Johnsen et al., 2020). In the morning tests, the spread of facing directions was tighter than in all the tests, as seen in the longer resultant vector. There was also a clearer pattern of facing in different directions after the first 2 s (Fig. S2). The confusing effects of daytime training may have dissipated overnight, allowing the ants’ memories to emerge more clearly at the start of the day.

### Is moving in the opposite direction to a goal a sign of uncertainty?

Ants in these experiments often moved toward a fictive goal in the opposite direction from the real goal. The ants’ uncertainty about the correct path to follow could be why they often faced or moved in precisely the opposite direction to the magnetically signalled route. Both directions frequently occurred in the same path (Fig. 3). While switching direction could reflect the ants’ uncertainty, it is a common occurrence in insect navigation, with several uses. First, honeybees and ants often invert the direction of route vectors between their nest and a food site. A switch in route direction can be induced by just starving or feeding bees (Dyer et al., 2002) or ants (Harris et al., 2005). Ants also look back to face the nest when first learning a route, thus enabling them to memorise the views along both directions when following a route (Graham and Collett, 2006; Schwarz et al., 2017, 2020).

In sum, while we are confident that ants were unsure of the correct direction to take, we have little direct evidence of a link between their uncertainty and their tendency to face in both directions. One indication is facing toward the fictive goal when room cues are made prominent (Fig. 6). Another comes from the behaviour of ants when training is reinforced with a black bar (Fig. 2 and Fig. S1). In these training trials, a vertical bar was fixed above the food. Ants tended to move directly away from the bar toward the start of their path. The bar, as it was not always present during training, may have made them hesitant that it was correct to move in the food direction.

Ants were also unsure what to do in the second set of experiments. We suggest that turning away from the current direction may occur because ants are uncertain about the correct destination. The undecided ant may not know the location of the triangle that it is not currently approaching. By turning away, it unlocks its attention and frees itself to switch its path directly towards the other triangle (Fig. 7C). This kind of behaviour was first described decades ago in walking, wingless *Drosophila.* The flies, when confined to a small arena with two inaccessible visual targets, 180 deg apart, oscillated between them for several hours, like Buridan's fictional ass trying vainly to choose between two identical piles of hay (Bülthoff et al., 1982). Were there no immediate alternative to capture the insect's attention, would ants turn in the diametrically opposite direction, as they did in the first set of experiments?

In sum, these experiments demonstrate that ants can learn to face in a magnetically defined direction and that their memory of the direction persists overnight. There is suggestive evidence that path integration can work with magnetic cues. The ants also showed uncertainty in their behaviour. Their indecision could be a consequence of the small area in which the experiments were conducted. It could also arise from competition between magnetic and other directional cues.

## Supplementary Material

Supplementary information
