# Supplementary material for: Wood ants learn the magnetic direction of a route but express uncertainty because of competing directional cues
Source: J Exp Biol. 2022 Aug 26;225(16):jeb244416. doi: 10.1242/jeb.244416 (PMC9482151; doi:10.1242/jeb.244416)
Supplement: Supplementary information [file jexbio-225-244416-s1.pdf]

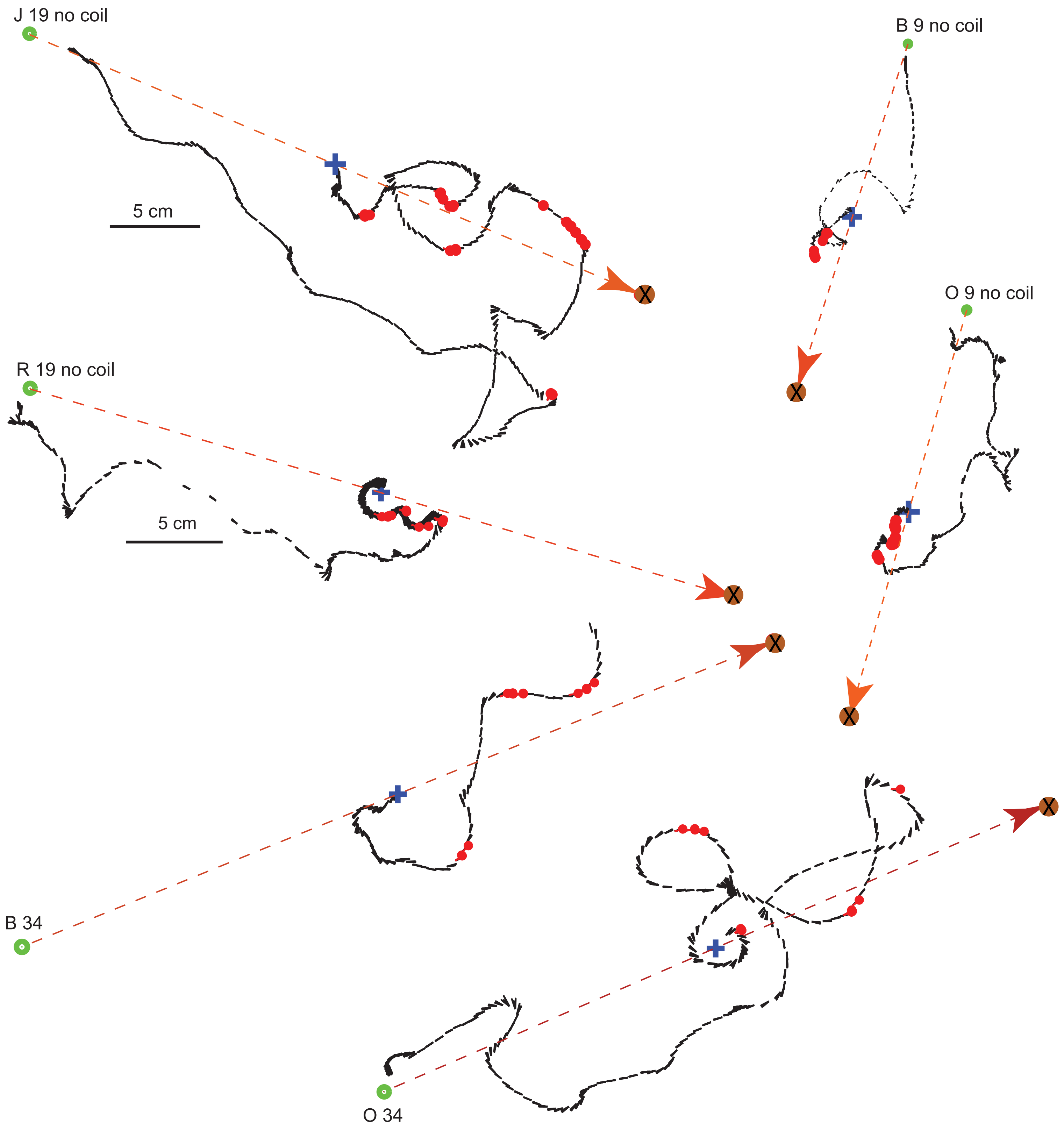

Fig. S1. More training trials with a black stripe above goal. Blue cross marks starting point. Red disks indicate frames in which the ant faces (within ±10 deg) of a fictive goal (coloured disk with an X) diametrically opposite to food. Real and fictive goals are both 18 cm from the central starting point. Letters and numbers indicate identity of ant and training trial. ‘No coil’ indicates magnetic coil was inactive. The coil was active on trial 34.

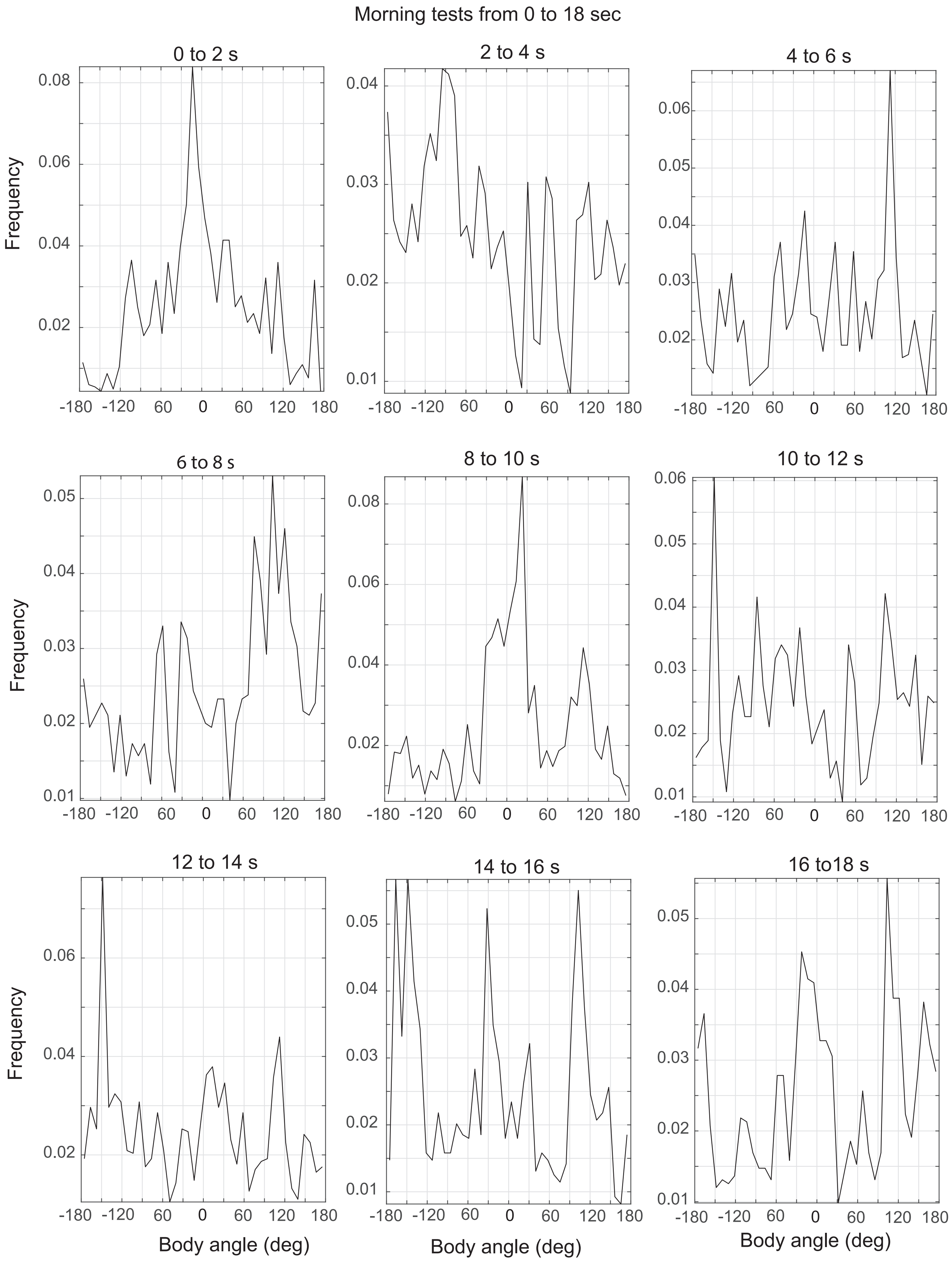

Fig. S2. Body angles relative to coil West in early morning tests. Plot shows the distribution of body angles in successive 2 sec intervals from 0 to 18 sec.

Journal of Experimental Biology • Supplementary information

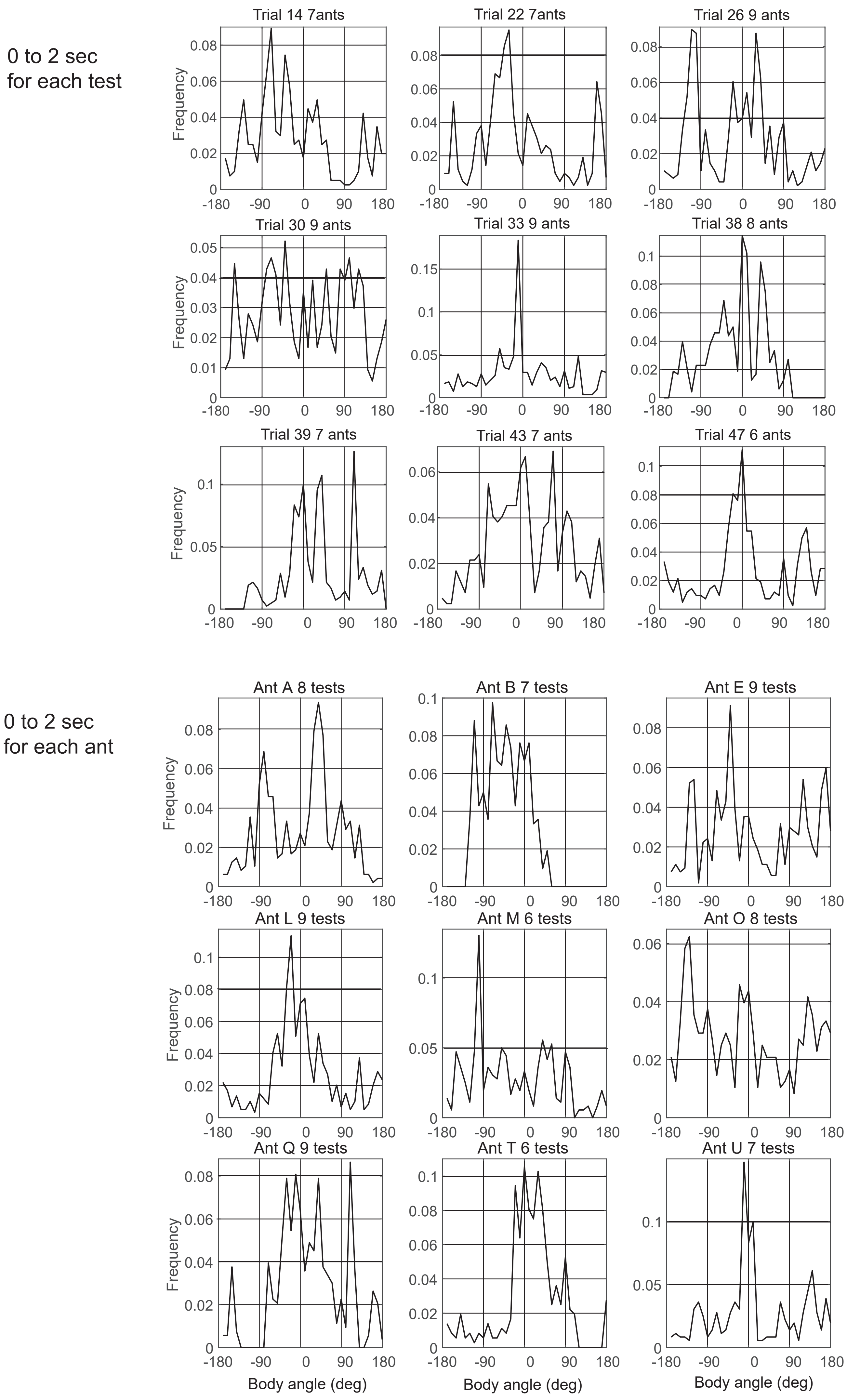

Fig. S3. Distributions of body angles during 0 to 2 sec aggregated over each test and each ant.

Journal of Experimental Biology • Supplementary information
